# Supplementary material for: A multiplex chemiluminescent immunoassay for serological profiling of COVID-19-positive symptomatic and asymptomatic patients
Source: Nat Commun. 2021 Feb 2;12:740. doi: 10.1038/s41467-021-21040-7 (PMC7854643; doi:10.1038/s41467-021-21040-7)
Supplement: Supplementary file 1 — Supplementary Information [file 41467_2021_21040_MOESM1_ESM.pdf]

Supplementary Information

A multiplex chemiluminescent immunoassay for serological profiling of COVID-19-positive symptomatic and asymptomatic patients

Allison N. Grossberg and Daniel A. Linseman

Supplementary Table 1. Reported symptoms and range of symptom severity in symptomatic COVID-19-positive and symptomatic COVID-19-negative participants.

	COVID-19-Positive	COVID-19-Negative	*p*-Value
**Number of participants reporting symptoms**	21	18	-
**Days between symptom onset and symptom resolution**	24.1 (4.63) [3 - 74]	16.5 (6.07) [1 - 102]	p = 0.32
**Fever**	13 (61.9%)<br>2.46 (0.35) [1 - 5]	15 (83.3%)<br>2.20 (0.30) [1 - 4]	p = 0.57
**Dry cough**	15 (71.4%)<br>3.20 (0.33) [1 - 5]	17 (94.4%)<br>2.82 (0.29) [1 - 5]	p = 0.39
**Sore throat**	11 (52.4%)<br>2.45 (0.37) [1 - 4]	17 (94.4%)<br>2.76 (0.38) [1 - 5]	p = 0.58
**Fatigue**	19 (90.5%)<br>3.37 (0.24) [1 - 5]	17 (94.4%)<br>3.06 (0.28) [1 - 5]	p = 0.41
**Sputum production**	12 (57.1%)<br>2.33 (0.40) [1 - 5]	13 (72.2%)<br>2.62 (0.33) [1 - 4]	p = 0.59
**Nasal congestion**	12 (57.1%)<br>3.00 (0.41) [1 - 5]	16 (88.9%)<br>2.88 (0.27) [1 - 4]	p = 0.79
**Runny nose**	13 (61.9%)<br>2.46 (0.42) [1 - 5]	18 (100.0%)<br>2.50 (0.28) [1 - 5]	p = 0.94
**Headache**	15 (71.4%)<br>2.87 (0.26) [1 - 5]	16 (88.9%)<br>2.50 (0.30) [1 - 5]	p = 0.37
**Loss of taste**	6 (28.6%)<br>3.50 (0.72) [1 - 5]	4 (22.2%)<br>2.00 (0.41) [1 - 3]	p = 0.15
**Loss of smell**	4 (19.0%)<br>4.50 (0.50) [3 - 5]	4 (22.2%)<br>2.50 (0.65) [1 - 4]	**p = 0.05**
**Vomiting**	3 (14.3%)<br>2.33 (0.67) [1 - 3]	1 (5.6%)<br>2.00 (NA) [2 - 2]	p = 0.83
**Diarrhea**	13 (61.9%)<br>2.38 (0.38) [1 - 5]	11 (61.1%)<br>2.27 (0.30) [1 - 4]	p = 0.83
**Dizziness**	9 (42.9%)<br>2.11 (0.31) [1 - 4]	6 (33.3%)<br>2.00 (0.37) [1 - 3]	p = 0.82
**Chills**	14 (66.7%)<br>2.86 (0.33) [1 - 5]	13 (72.2%)<br>2.69 (0.40) [1 - 5]	p = 0.75
**Body aches or myalgia**	14 (66.7%)<br>2.86 (0.35) [1 - 5]	15 (83.3%)<br>2.93 (0.25) [1 - 4]	p = 0.86
**Shortness of breath**	12 (57.1%)<br>2.25 (0.39) [1 - 5]	9 (50.0%)<br>2.44 (0.47) [1 - 4]	p = 0.75
**Swollen lymph nodes**	4 (19.0%)<br>2.20 (0.73) [1 - 5]	1 (5.6%)<br>3.00 (0.58) [2 - 4]	p = 0.48
**Chest pain**	8 (38.1%)<br>2.63 (0.32) [1 - 4]	7 (38.9%)<br>2.00 (0.44) [1 - 4]	p = 0.26

Days between symptom onset and symptom resolution are also shown for comparison for symptomatic COVID-19-positive (n=21) and -negative (n=18) participants. Data are presented as the mean (SE), [range], or number (%). *p*-values were determined via two-tailed Independent Samples T-Tests.

Supplementary Table 2. Analysis of variance table comparing general additive models (GAM) and general linear models (GLM) for IgG average, IgM average, and IgM titers against SARS-CoV-2 spike 1 glycoprotein (S1 SP) and receptor binding domain (RBD) as a function of days between symptom onset and initial test.

Antibody/Antigen	Model	Res.Df	RSS	Df	Sum of Sq	*p* value
**IgG Average – Figure 2a**	GLM	19.00	5.09			
	GAM	16.87	3.59	2.13	1.51	*p* =0.03
**IgM Average – Figure 2b**	GLM	19.00	2.17			
	GAM	18.31	1.96	0.69	0.21	*p*=0.09
**IgM S1 SP – Figure 2c**	GLM	19.00	4.48			
	GAM	17.59	3.23	1.41	1.25	*p*=0.02
**IgM RBD – Figure 2d**	GLM	19.00	4.47			
	GAM	15.87	2.50	3.13	1.97	*p*=0.01

An ANOVA was used to compare each GAM model to a GLM model with the same terms in order to determine whether the GAM model was significantly better at capturing the data than the GLM model. Compared to the GLM models, the GAM models were a better fit of the data presented in Figure 2a, 2c and 2d.

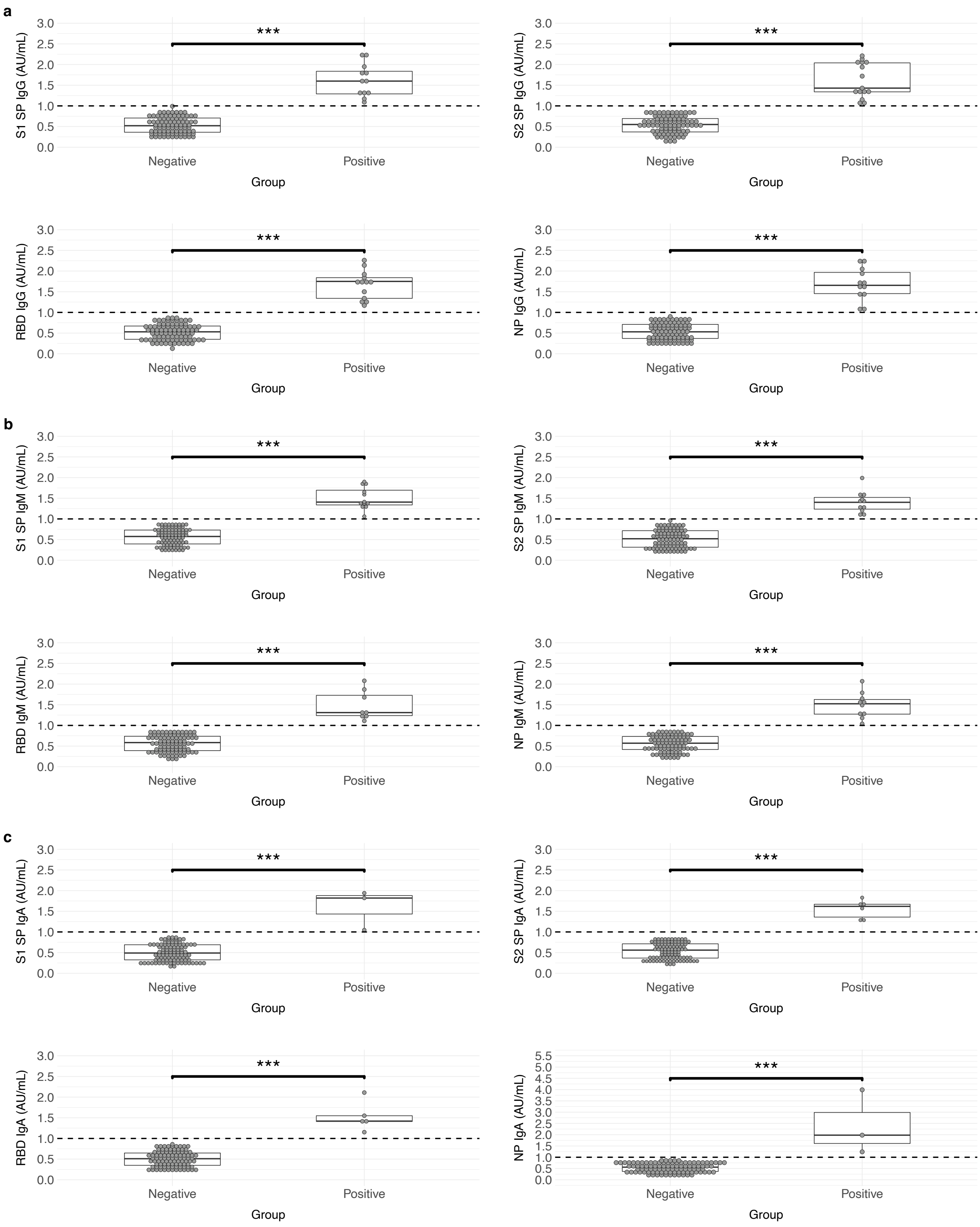

Supplementary Figure 1. Comparison of titers in all participants positive (pos) and negative (neg) for IgA, IgG, and IgM antibodies against SARS-CoV-2 antigens. a) Titers are displayed for all participants positive (titers ≥1.0) and negative for IgG antibodies against spike 1 glycoprotein (S1 SP; pos: n=12, neg: n=90), spike 2 glycoprotein (S2 SP; pos: n=15, neg: n=87), receptor binding domain (RBD; pos: n=13, neg: n=89), and nucleoprotein (NP; pos: n=12, neg: n=90). For all IgG antigens, COVID-19-positive participants had significantly higher titers than negative participants ($p<2.2 \times 10^{-16}$). **b)** Titers are displayed for all participants positive and negative for IgM antibodies against S1 SP (pos: n=12, neg: n=90), S2 SP (pos: n=11, neg: n=91), RBD (pos: n=8, neg: n=94), and NP (pos: n=10, neg: n=92). For all IgM antigens, COVID-19-positive participants had significantly higher titers than negative participants ($p<2.2 \times 10^{-16}$). **c)** Titers are displayed for all participants positive and negative for IgA antibodies against S1 SP (pos: n=3, neg: n=99), S2 SP (pos: n=6, neg: n=96), RBD (pos: n=5, neg: n=97), and NP (pos: n=3, neg: n=99). COVID-19-positive participants had significantly higher titers than negative participants for S1 SP IgA ($p=2.4 \times 10^{-14}$) and for all other IgA antigens ($p<2.2 \times 10^{-16}$). The cut-off value for the serological test (≥1) is shown as a black dashed line for reference. Boxplots represent the minimum, maximum, median, first quartile and third quartile in the data set. All data were analyzed using two-sided Independent Samples T-Tests. *** indicates $p<0.001$.

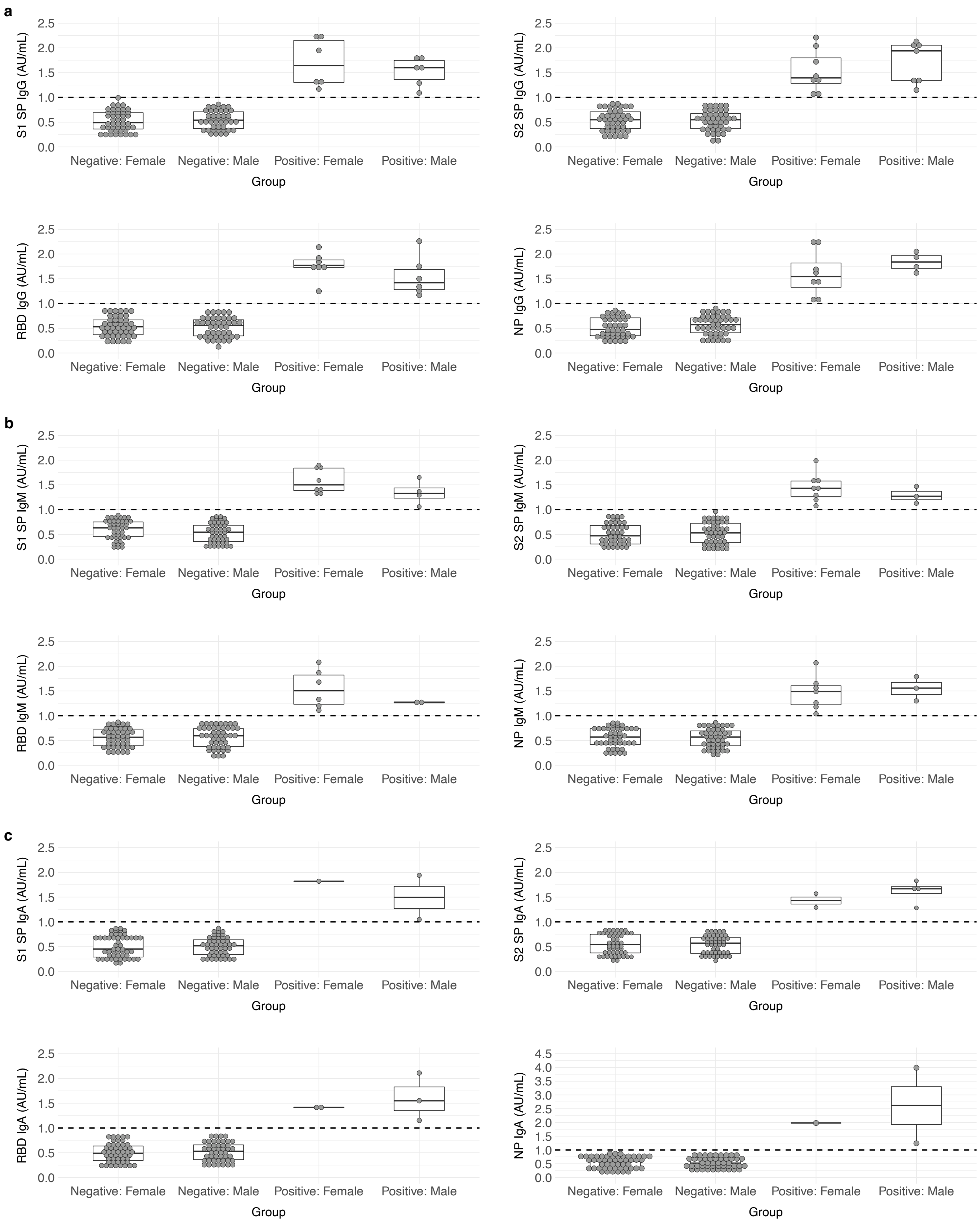

Supplementary Figure 2. Comparison of IgA, IgG, and IgM antibody titers against SARS-CoV-2 antigens in COVID-19-positive and -negative females and males. a) Titers are shown for COVID-19-negative females (NF) and males (NM) and –positive females (PF) and males (PM) for IgG antibodies against the spike 1 glycoprotein (S1 SP; NF: n=46, NM: n=44, PF: n=6, PM: n=6), spike 2 glycoprotein (S2 SP; NF: n=44, NM: n=43, PF: n=8, PM: n=7), receptor binding domain (RBD; NF: n=45, NM: n=44, PF: n=7, PM: n=6), and nucleoprotein (NP; NF: n=44, NM: n=46, PF: n=8, PM: n=4) . **b)** Titers are shown for IgM antibodies against the S1 SP (NF: n=44, NM: n=46, PF: n=8, PM: n=4), S2 SP (NF: n=44, NM: n=47, PF: n=8, PM: n=3), RBD (NF: n=46, NM: n=48, PF: n=6, PM: n=2), and NP (NF: n=45, NM: n=47, PF: n=7, PM: n=3). **c)** Titers are shown for IgA antibodies against the S1 SP (NF: n=51, NM: n=48, PF: n=1, PM: n=2), S2 SP (NF: n=50, NM: n=46, PF: n=2, PM: n=4), RBD (NF: n=50, NM: n=47, PF: n=2, PM: n=3), and NP (NF: n=51, NM: n=48, PF: n=1, PM: n=2). Titers are displayed only for COVID-19-positive males and females that were positive (titers ≥1) for each antibody against each antigen. Boxplots represent the minimum, maximum, median, first quartile and third quartile in the data set. The cut-off value for the serological test (≥1) is shown as a black dashed line for reference. All data were analyzed using two-sided Independent Samples T-Tests to compare COVID-19-negative females and males and COVID-19-positive females and males. No data were statistically significant.

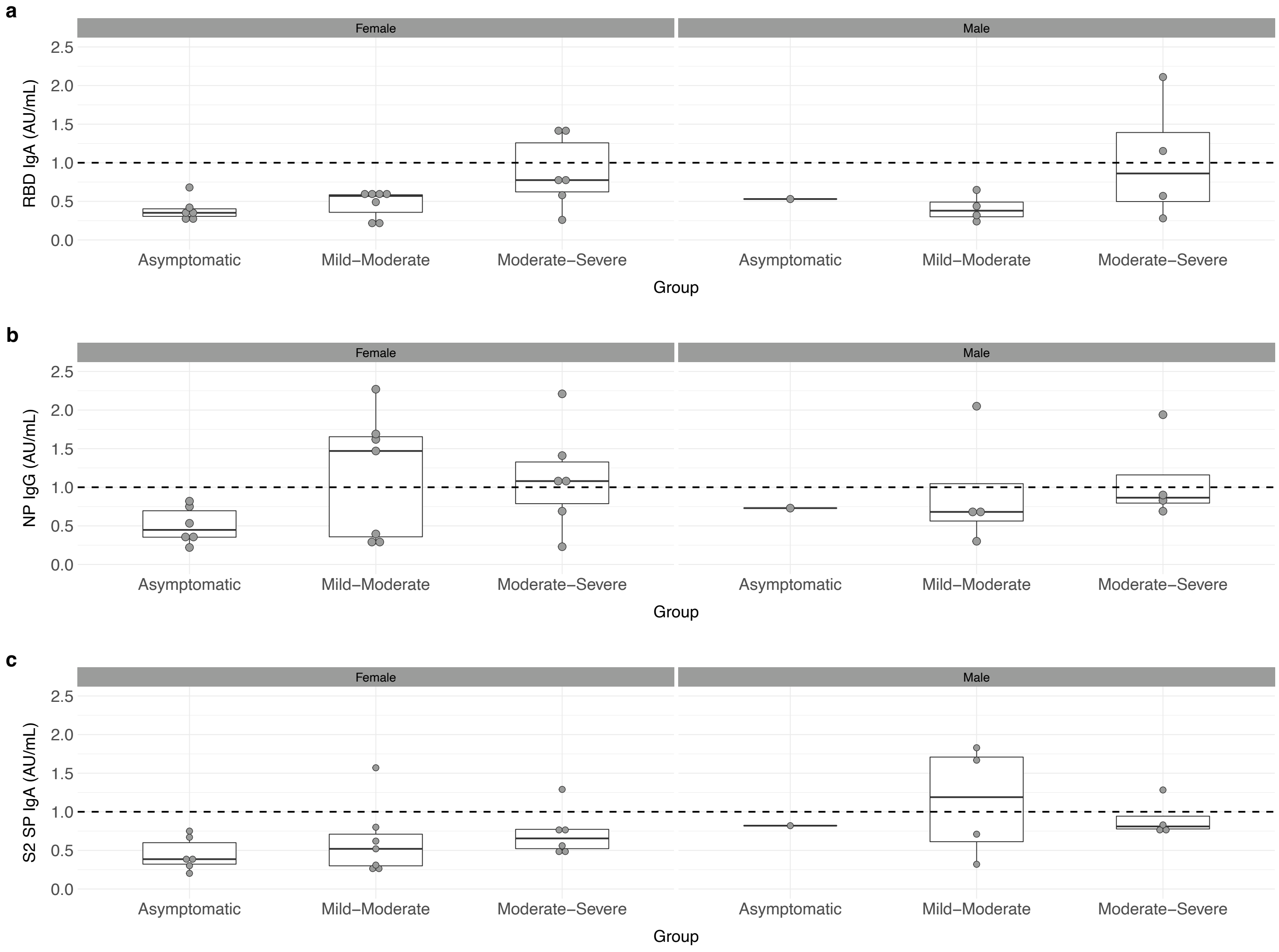

Supplementary Figure 3. Comparison of IgA antibody titers against receptor binding domain (RBD) and spike 2 glycoprotein (S2 SP) and IgG antibody titers against nucleoprotein (NP) in COVID-19-positive female and male participants with varying symptomology. Titers are shown for COVID-19-positive females and males who reported no symptoms (asymptomatic), mild-moderate, and moderate-severe symptoms for **a)** IgA antibodies against RBD (asymptomatic female: n=6, mild-moderate female: n=7, moderate-severe female: n=6, asymptomatic male: n=1, mild-moderate male: n=4, moderate-severe male: n=4) **b)** IgG antibodies against NP (asymptomatic female: n=6, mild-moderate female: n=7, moderate-severe female: n=6, asymptomatic male: n=1, mild-moderate male: n=4, moderate-severe male: n=4), and **c)** IgA antibodies against S2 SP (asymptomatic female: n=6, mild-moderate female: n=7, moderate-severe female: n=6, asymptomatic male: n=1, mild-moderate male: n=4, moderate-severe male: n=4). Boxplots represent the minimum, maximum, median, first quartile and third quartile in the data set. The cut-off value for the serological test (≥1) is shown as a black dashed line for reference. All data were analyzed using one-way ANOVA to compare female or male participants with varying symptom severity. No data were statistically significant.

a

b

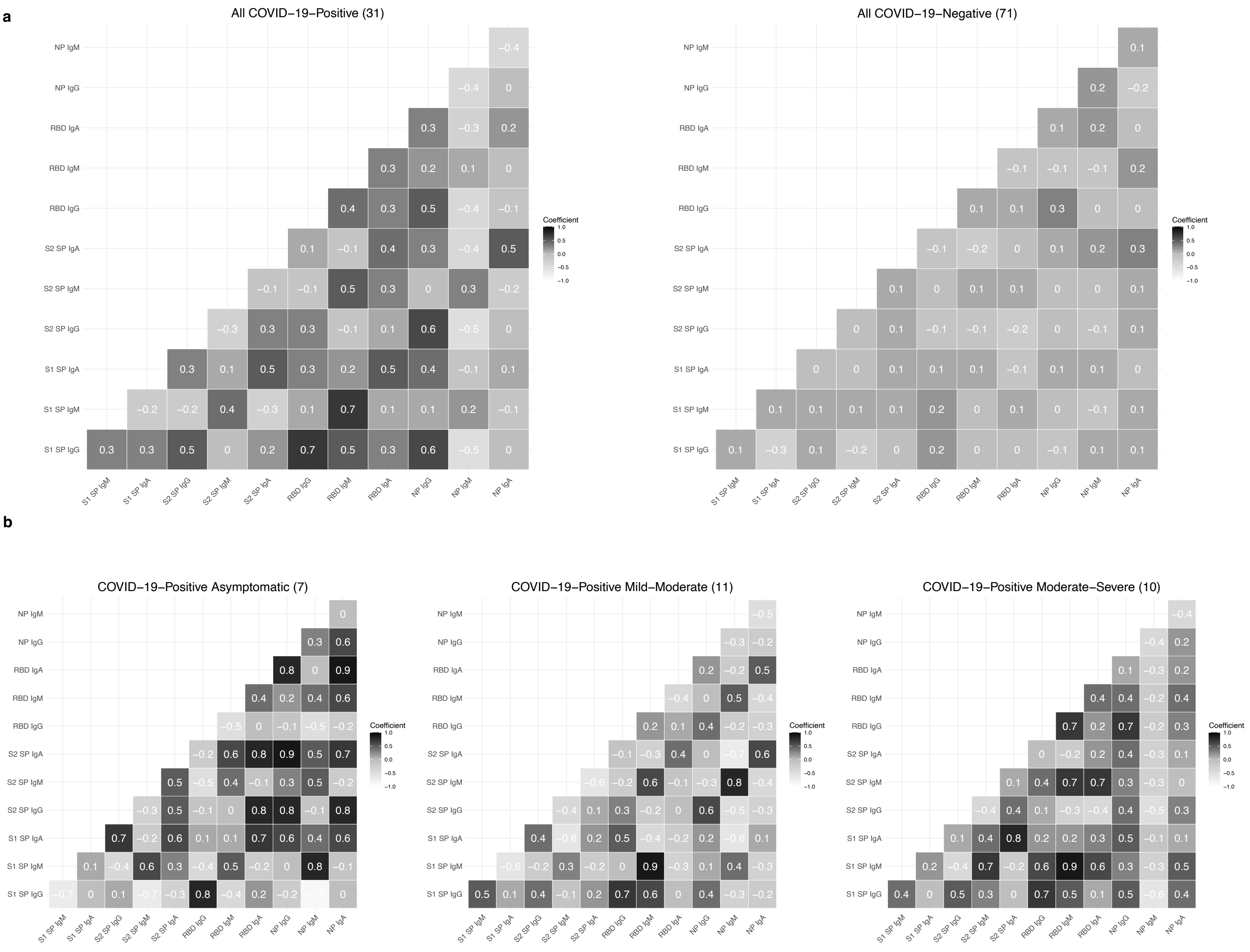

Supplementary Figure 4. Correlations between titers against SARS-CoV-2 antigens between COVID-19-positive versus –negative participants, and between COVID-19-positive participants with varied symptomology. a) Correlograms display Pearson correlations among IgM, IgA, and IgG titers against spike 1 glycoprotein (S1 SP), spike 2 glycoprotein (S2 SP), receptor binding domain (RBD), and nucleoprotein (NP) in COVID-19-positive (n=31) and -negative (n=71) participants. **b)** Correlograms display Pearson correlations among IgM, IgA, and IgG titers against S1 SP, S2 SP, RBD, and NP in COVID-19-positive asymptomatic participants (n=7), participants who reported mild-moderate symptoms (n=11), and participants who reported moderate-severe symptoms (n=10). Shading indicates whether the correlation is positive (black) or negative (white). The shade of squares indicates the strength of the correlation, with a darker shade indicating a stronger correlation and a lighter shade indicating weaker ones. Correlation coefficients are displayed in each square.

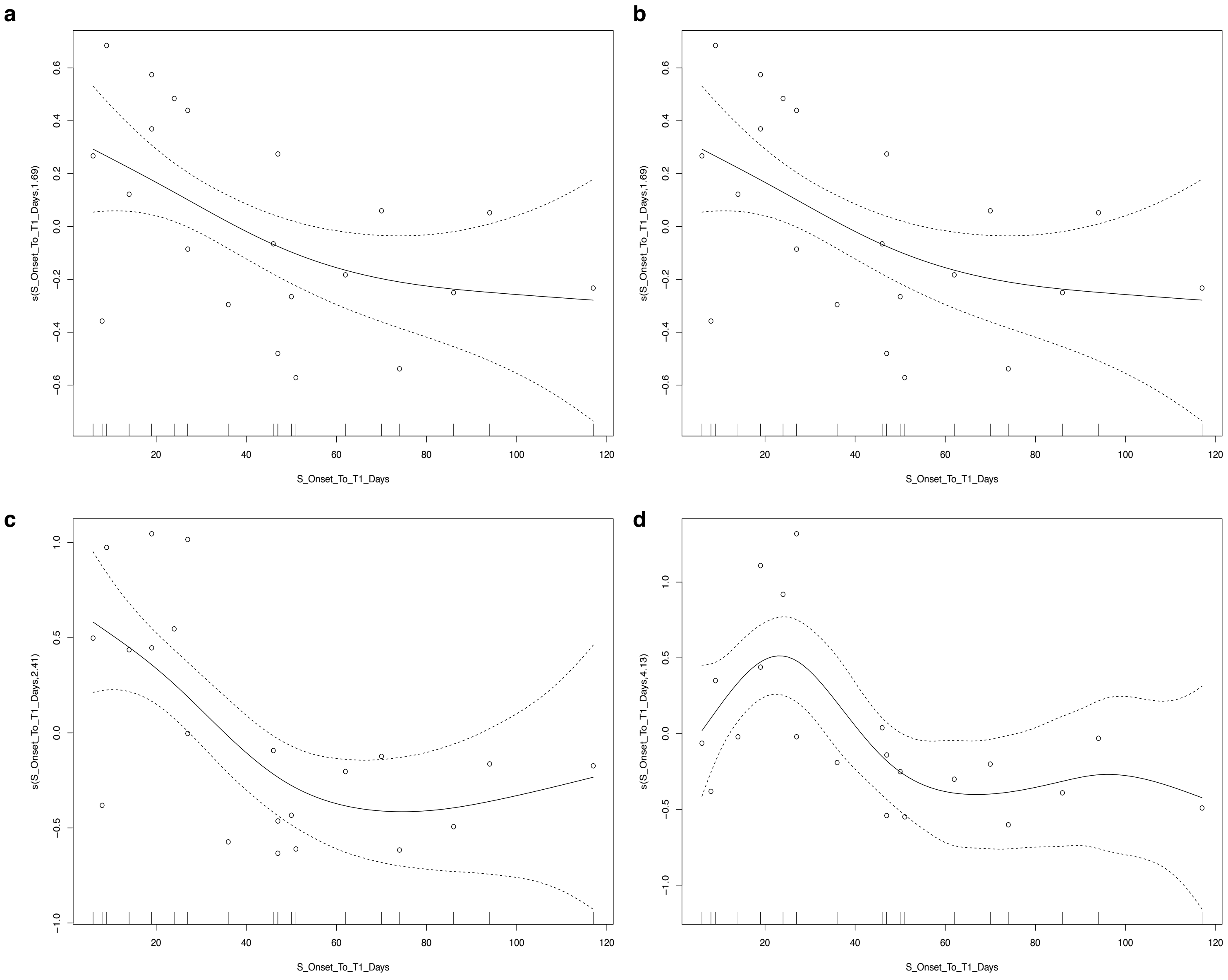

Supplementary Figure 5. Non-linear trend lines generated using general additive models (GAM) for IgG and IgM antibody titers against SARS-CoV-2 antigens as a function of days between symptom onset and serological test. White circles represent titers of **a)** IgG average, **b)** IgM average, and **c)** IgM antibodies against SARS-CoV-2 spike 1 glycoprotein (S1 SP) and **d)** receptor binding domain (RBD) in symptomatic COVID-19-positive participants (n=21) plotted against days between symptom onset and serological test. The solid line represents a GAM trendline showing the relationship between titers and days between symptom onset and serological test. The 95% confidence interval is displayed by the lower and upper black dotted lines.
