# Supplementary material for: A multiplex chemiluminescent immunoassay for serological profiling of COVID-19-positive symptomatic and asymptomatic patients
Source: Nat Commun. 2021 Feb 2;12:740. doi: 10.1038/s41467-021-21040-7 (PMC7854643; doi:10.1038/s41467-021-21040-7)
Supplement: Supplementary file 3 — Reporting Summary [file 41467_2021_21040_MOESM3_ESM.pdf]

nature research

Corresponding author(s): Linseman, Daniel A

Last updated by author(s): January 7, 2021

Reporting Summary

Nature Research wishes to improve the reproducibility of the work that we publish. This form provides structure for consistency and transparency in reporting. For further information on Nature Research policies, see our Editorial Policies and the Editorial Policy Checklist.

Statistics

For all statistical analyses, confirm that the following items are present in the figure legend, table legend, main text, or Methods section.

n/a	Confirmed	
☐	☒	The exact sample size (*n*) for each experimental group/condition, given as a discrete number and unit of measurement
☐	☒	A statement on whether measurements were taken from distinct samples or whether the same sample was measured repeatedly
☐	☒	The statistical test(s) used AND whether they are one- or two-sided<br>*Only common tests should be described solely by name; describe more complex techniques in the Methods section.*
☒	☐	A description of all covariates tested
☐	☒	A description of any assumptions or corrections, such as tests of normality and adjustment for multiple comparisons
☐	☒	A full description of the statistical parameters including central tendency (e.g. means) or other basic estimates (e.g. regression coefficient) AND variation (e.g. standard deviation) or associated estimates of uncertainty (e.g. confidence intervals)
☐	☒	For null hypothesis testing, the test statistic (e.g. *F*, *t*, *r*) with confidence intervals, effect sizes, degrees of freedom and *P* value noted<br>*Give P values as exact values whenever suitable.*
☒	☐	For Bayesian analysis, information on the choice of priors and Markov chain Monte Carlo settings
☒	☐	For hierarchical and complex designs, identification of the appropriate level for tests and full reporting of outcomes
☐	☒	Estimates of effect sizes (e.g. Cohen's *d*, Pearson's *r*), indicating how they were calculated

Our web collection on statistics for biologists contains articles on many of the points above.

Software and code

Policy information about availability of computer code

Data collection	Data were collected using Microsoft Excel (version number 16.44) for Mac 16.36 and Office 365 2020. Patient samples were analyzed by Vibrant Clinical Labs. The intensity of the signal from each chip in the CLIA assay was measured using a high resolution chemiluminescence imager (Q-View™ Imager Pro, Quansys Biosciences, Logan UT). The raw chemiluminescent signals for all probes were extracted from the images using Vibrant's in-house reporter software. The software does not have an associated version number to report.
Data analysis	All statistical analyses and data visualization were performed using RStudio (version number 1.3.1093) for Mac (RStudio Team [2019]. RStudio: Integrated Development for R. RStudio, Inc., Boston, MA URL). Vibrant analyzed the raw chemiluminescent signals from the CLIA assay using an in-house software. The raw data were subjected to quantile normalization, spatial correction, and background correction. Custom R code has been deposited in a GitHub repository and the link has been provided under the code availability section. https://github.com/linsemanlab/Grossberg-Koza-et-al-2020.

For manuscripts utilizing custom algorithms or software that are central to the research but not yet described in published literature, software must be made available to editors and reviewers. We strongly encourage code deposition in a community repository (e.g. GitHub). See the Nature Research guidelines for submitting code & software for further information.

Data

Policy information about availability of data

All manuscripts must include a data availability statement. This statement should provide the following information, where applicable:

- Accession codes, unique identifiers, or web links for publicly available datasets
- A list of figures that have associated raw data
- A description of any restrictions on data availability

The data availability statement has been updated. All source data (including data for each figure and raw serological data) has been uploaded to a public GitHub repository.

Field-specific reporting

Please select the one below that is the best fit for your research. If you are not sure, read the appropriate sections before making your selection.

☒ Life sciences ☐ Behavioural & social sciences ☐ Ecological, evolutionary & environmental sciences

For a reference copy of the document with all sections, see nature.com/documents/nr-reporting-summary-flat.pdf

Life sciences study design

All studies must disclose on these points even when the disclosure is negative.

Sample size	This study included one hundred seven non-hospitalized participants (n=107) who we were able to recruit from a community clinic designated for the care of patients without severe or life-threatening symptoms of COVID-19 between March and May 2020.
Data exclusions	Participants were excluded from the study if they were not permanent residents of Colorado or if they reported symptoms before December 25, 2019. Exclusion criteria were not pre-established.
Replication	Replication was not relevant because data was collected from human research subjects who underwent a blood draw at a single time point.
Randomization	Randomization was not relevant because the participants did not undergo any treatment or experimental protocol. All data were de-identified and analyzed retrospectively.
Blinding	Blinding was not relevant because the participants did not undergo any treatment or experimental manipulation. All data were de-identified and analyzed retrospectively.

Reporting for specific materials, systems and methods

We require information from authors about some types of materials, experimental systems and methods used in many studies. Here, indicate whether each material, system or method listed is relevant to your study. If you are not sure if a list item applies to your research, read the appropriate section before selecting a response.

Materials & experimental systems

n/a	Involved in the study	
☐	☒	Antibodies
☒	☐	Eukaryotic cell lines
☒	☐	Palaeontology and archaeology
☒	☐	Animals and other organisms
☒	☐	Human research participants
☒	☐	Clinical data
☒	☐	Dual use research of concern

Methods

n/a	Involved in the study	
☒	☐	ChIP-seq
☒	☐	Flow cytometry
☒	☐	MRI-based neuroimaging

Antibodies

Antibodies used	Invitrogen - Goat anti-Human IgG Fc Highly Cross-Adsorbed Secondary Antibody, HRP - A18829<br>Life Tech - Goat anti-Human IgM (u chain), HRP Conjugate - A18841<br>Invitrogen - Goat Anti-Human IgA (Heavy Chain) Secondary Antibody HRP - 31417
Validation	Antibodies used in the CLIA assay performed by Vibrant Clinical Labs are described here (unpublished pre-print: https://www.medrxiv.org/content/10.1101/2020.04.29.20085068v1).
